# Preclinical Antimalarial Combination Study of M5717, a Plasmodium falciparum Elongation Factor 2 Inhibitor, and Pyronaridine, a Hemozoin Formation Inhibitor

**DOI:** 10.1128/AAC.02181-19

**Published:** 2020-03-24

**Authors:** Matthias Rottmann, Brian Jonat, Christin Gumpp, Satish K. Dhingra, Marla J. Giddins, Xiaoyan Yin, Lassina Badolo, Beatrice Greco, David A. Fidock, Claude Oeuvray, Thomas Spangenberg

**Affiliations:** a Department of Medical Parasitology and Infection Biology, Swiss Tropical and Public Health Institute, Basel, Switzerland; b University of Basel, Basel, Switzerland; c Department of Pediatrics, Columbia University Irving Medical Center, New York, New York, USA; d Department of Microbiology and Immunology, Columbia University Irving Medical Center, New York, New York, USA; e Global Statistics for NDD, Immunology, Endocrinology, Fertility & Others, EMD Serono, Billerica, Massachusetts, USA; f Discovery and Development Technologies, Merck Healthcare KGaA, Darmstadt, Germany; g Global Health Institute of Merck, Eysins, Switzerland; h Division of Infectious Diseases, Department of Medicine, Columbia University Irving Medical Center, New York, New York, USA

**Keywords:** M5717, *Plasmodium falciparum*, SCID mouse, drug combination, isobologram, malaria, pyronaridine, resistant mutant

## Abstract

Antimalarial drug resistance in the Plasmodium falciparum parasite poses a constant challenge for drug development. To mitigate this risk, new antimalarial medicines should be developed as fixed-dose combinations. Assessing the pharmacodynamic interactions of potential antimalarial drug combination partners during early phases of development is essential in developing the targeted parasitological and clinical profile of the final drug product. Here, we have studied the combination of M5717, a P. falciparum translation elongation factor 2 inhibitor, and pyronaridine, an inhibitor of hemozoin formation. Our test cascade consisted of *in vitro* isobolograms as well as *in vivo* studies in the P. falciparum severe combined immunodeficient (SCID) mouse model. We also analyzed pharmacokinetic and pharmacodynamic parameters, including genomic sequencing of recrudescent parasites. We observed no pharmacokinetic interactions with the combination of M5717 and pyronaridine. M5717 did not negatively impact the rate of kill of the faster-acting pyronaridine, and the latter was able to suppress the selection of M5717-resistant mutants, as well as significantly delay the recrudescence of parasites both with suboptimal and optimal dosing regimens.

## Abstract (precis)

Antimalarial drug resistance in the Plasmodium falciparum parasite poses a constant challenge for drug development. To mitigate this risk, new antimalarial medicines should be developed as fixed-dose combinations. Assessing the pharmacodynamic interactions of potential antimalarial drug combination partners during early phases of development is essential in developing the targeted parasitological and clinical profile of the final drug product. Here, we have studied the combination of M5717, a P. falciparum translation elongation factor 2 inhibitor, and pyronaridine, an inhibitor of hemozoin formation.

## INTRODUCTION

Antimalarial drug resistance in Plasmodium falciparum has led to the demise of multiple first-line treatments, including chloroquine, proguanil, pyrimethamine, sulfadoxine-pyrimethamine, mefloquine, and more recently the artemisinin-based combination therapy dihydroartemisinin-piperaquine, which was recently reported to produce only a 50% adequate clinical and parasitological response in sites across the Greater Mekong subregion (1–3).

To mitigate the risk of resistance, new antimalarial drugs should be developed as fixed-dose combinations. The rationale for combination therapy is that any parasite resistant to one component should be eliminated by the other, provided that both have distinct modes of action. This strategy is expected to significantly reduce the emergence of resistance (4). However, other factors that influence the emergence of drug resistance must be taken into account, including drug exposure, drug half-life, effects of the drug on other parasite life-cycle stages such as gametocytogenesis and gametocyte viability, clinical parasite reduction ratio, and drug dosage (5, 6).

**FIG 1:**
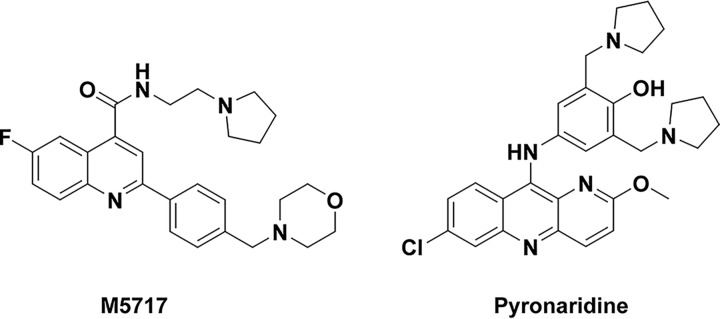
Chemical structures of M5717 and pyronaridine.

Recently, a quinoline-4-carboxamide antiplasmodial series was optimized to deliver lead molecules with low nanomolar *in vitro* potency and excellent oral efficacy in the Plasmodium berghei malaria mouse model with ED_90_ values below 1 mg/kg when dosed orally for 4 days (7, 8). The favorable efficacy, potency, selectivity, drug metabolism and pharmacokinetic (DMPK) properties, coupled with a novel mechanism of action, namely inhibition of P. falciparum translation elongation factor 2 (*PfeEF2*), led to the progression of M5717 (also known as DDD107498) from preclinical to clinical development (Fig. 1) (7, 8). The mean half-life (t_1/2_) ranged from 155 h to 193 h across doses in human volunteers. From a parasitological standpoint, M5717 is potent across the parasite life cycle. Due to its mode of action, the anti-parasitological activity is characterized by a rapid arrest of parasite growth with a delayed clearance of parasites from circulation, while also acting on liver stages and gametocytes. This multistage activity provides additional benefits for treatment, such as prophylaxis or transmission-blocking potential (9). Not surprisingly, at clinically relevant parasite inoculum levels, M5717-resistant parasites can be selected *in vitro* and lead to various degrees of susceptibility, highlighting the need for a well-selected partner drug during the combination phases (7).

With this in mind, we selected pyronaridine (10–15), a benzonaphthyridine derivative discovered in China in the late 1970s, for partnering with M5717. Pyronaridine fulfilled three criteria that seemed to provide reasonable prerequisites for the combination strategy (Fig. 1): (i) matching half-lives to avoid periods where only one drug achieves therapeutic levels, where pyronaridine is a long-lasting drug with a t_1/2_ of 241 h at 400 mg in healthy volunteers (16); (ii) complementary modes of action in regard to the rate of killing, where pyronaridine is a fast-acting (chloroquine-like) drug that has the demonstrated ability to inhibit β-hematin formation *in vitro* (17, 18); and (iii) a lack of resistance reported to date in P. falciparum (19).

Here, we describe the pharmacokinetic and pharmacodynamic properties of the combination of M5717 and pyronaridine, both *in vitro* through asexual blood-stage P. falciparum parasites as well as *in vivo* by means of the well-established P. falciparum SCID mouse model (20). The objective of the studies was to evaluate and describe the parasitological behavior and pharmacokinetic parameters of the two compounds when used in combination.

## RESULTS

### *In vitro* drug interactions.

We observed no interaction between M5717 and pyronaridine with our *in vitro* isobologram experiments with three different assay durations (Table 1; see Materials and Methods). Experimental half maximal effective concentration (EC_50_) values (M5717, 0.3 nM; pyronaridine, 3.5 nM; atovaquone, 0.4 nM; proguanil, 5.5 μM; [^3^H]hypoxanthine incorporation, 72 h, NF54 strain) of P. falciparum were in close agreement with the literature (21), and we observed the sum of the fractional 50% inhibitory concentration (ΣFIC_50_) values in the range of 1.3 to 1.7 from three independent experiments for this combination conducted for 48 and 72 h. These results suggested that the interactions between M5717 and pyronaridine were not detrimental, but rather were additive under these test conditions. Control experiments with the previously described synergistic drug combination of atovaquone + proguanil showed the expected level of synergy, with ΣFIC_50_ values in the range of 0.2 to 0.4 across 48 h and 72 h assays (Table 1) (22).

**TABLE 1:** *In vitro* drug combination assays for M5717+pyronaridine and atovaquone+proguanil in the NF54 strain^*a*^^,^^*b*^

Drug partners	ΣFIC_50_ at 48 h	ΣFIC_50_ at 72 h	Interaction at 72 h	Combination ratio	Drug partners	ΣFIC_50_ at 48 h	ΣFIC_50_ at 72 h	Interaction at 72 h
M5717 + PYRO	1.7 ± 0.12	1.4 ± 0.12	Nondetrimental interaction	1 + 3	ATO + PRO	0.4 ± 0.04	0.3 ± 0.08	Synergistic
M5717 + PYRO	1.5 ± 0.38	1.4 ± 0.26	Nondetrimental interaction	1 + 1	ATO + PRO	0.3 ± 0.06	0.3 ± 0.05	Synergistic
M5717 + PYRO	1.3 ± 0.17	1.4 ± 017	Nondetrimental interaction	3 + 1	ATO + PRO	0.3 ± 0.03	0.2 ± 0.05	Synergistic

a ATO, atovaquone; PRO, proguanil; PYRO, pyronaridine.

b ΣFIC_50_ (fractional inhibitory concentrations) indicate the following: synergism at ΣFIC_50_ ≤0.5; antagonism at ΣFIC_50_ >2.0; nondetrimental interactions when 0.5 < ƩFIC_50_ ≤2.0. The values show the mean of 3 independent assays for NF54.

### *In vivo* combination.

We observed no detrimental pharmacodynamic and pharmacokinetic interactions between M5717 and pyronaridine with our *in vivo* combination experiments (Table 2; see Materials and Methods).

**TABLE 2:** Pharmacodynamic (PD) and pharmacokinetic (PK) properties of M5717, pyronaridine, and M5717 + pyronaridine combination *in vivo* against P. falciparum Pf3D70087/N9 administered as a single oral dose

Entry	M5717 exposure		Pyronaridine exposure		Parasitemia^*b*^					
	Dose (mg/kg)	AUC_0-inf_^*a*^ (ng · h/ml)	Dose (mg/kg)	AUC_0-inf_^*a*^ (ng · h/ml)	Day 3	Day 4	Day 5	Day 6	Mean DoR^*c*^	*n*
A	0	Not measured	0	Not measured	1.48	2.53	7.00	9.00	3	4
B	0	Not measured	0	Not measured	0.85	1.91	3.34	6.16	3	4
1	3	1,570 ± 139	0	Not measured	1.2	0.65	0.49	0.19	6	2
2	6	3,390 ± 479	0	Not measured	1.26	1.4	1.03	0.46	17	2
3	12	6,160 ± 454	0	Not measured	0.81	0.58	0.53	0.11	24	4
4	30	16,900 ± 199	0	Not measured	0.89	0.73	0.54	0.21	24	2
5	0	Not measured	6	6,560 ± 381	0.89	0.09	0.01	0.00^*d*^	24 to 35	2
6	0	Not measured	12	14,500 ± 1,060	1.51	0.13	0.00^*d*^	0.00^*d*^	35	2
7	0	Not measured	36	55,600 ± 2,080	1.61	0.12	0.00^*d*^	0.00^*d*^	≥60^*e*^	2
8	3	1,360 ± 94	6	8,500 ± 661	1.25	0.61	0.03	0.00^*d*^	35	2
9	12	6,400 ± 257	6	8,490 ± 2,260	0.83	0.34	0.03	0.04	45	3
10	12	6,650 ± 645	12	12,100 ± 1,820	0.83	0.21	0.01	0.00^*d*^	44	3
11	6	2,980±118	36	58,200 ± 193	1.29	0.24	0.03	0.00^*d*^	≥60^*e*^	2

a The AUCs are mean values ± SD with *n* ≥ 2.

b Mean % parasitemia as assessed by microscopy.

c DoR, day of recrudescence, uncorrected (days postinfection; treatment was made on day 3).

d Reached the lower limit of quantification (LLQ <0.01% parasitemia).

e Experiment ended at day 60 with parasitemia <0.01%.

When administered alone, M5717 displayed a half-life (t_1/2_) of ∼37 h (Fig. S1 in the supplemental material). M5717 showed a dose proportionality in exposure (AUC_0-inf_) at 3, 6, 12, and 30 mg/kg, with a parasite clearance time in line with previously described experiments, i.e., we observed a biphasic clearance of parasitemia with a breakpoint at around 48 h between both phases. The first phase was characterized by a low parasite reduction ratio (PRR) and a long half-life of circulating parasites, while the second phase showed a rapid decline in parasitemia with higher PRR and a shorter half-life (entries 1 to 4) (7). Parasite recrudescence could be extended until day 24 with a plateau between 12 and 30 mg/kg. Sequencing of parasites recrudescing from the 30 mg/kg dose group indicated mutation of amino acids 182 and 138 in the *eEF2* site (Table S4).

Pyronaridine dosed at 6, 12, and 36 mg/kg showed a good dose linearity and led to a rapid decline in parasitemia in 2 days (entries 5 to 7). With a dose of 36 mg/kg, there was no parasite recrudescence as of day 60, when the experiment was concluded. The t_1/2_ of pyronaridine was estimated at 74 h (Fig. S1).

In combination with pyronaridine, the pharmacokinetics of M5717 (*C*_max_, AUC_0-inf_, and t_1/2_) were unchanged compared to M5717 administered alone (entries 8 to 11). No change was observed in pyronaridine pharmacokinetics in the combination versus the single-agent treatments. Also, there was no relevant effect of M5717 on the rate of kill of pyronaridine at higher doses such as 12 and 36 mg/kg (entries 10 and 11).

**FIG 2:**
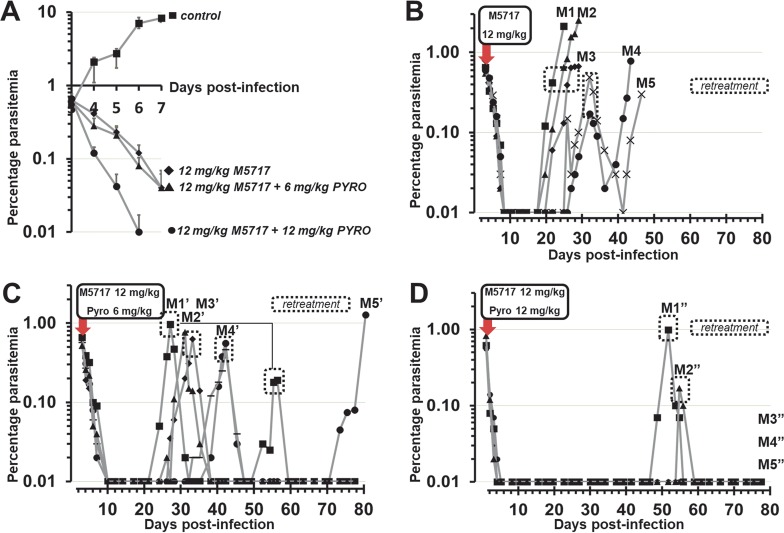
Pharmacodynamics upon single oral treatment of M5717 and pyronaridine (Pyro) as single agents or in combination against P. falciparum Pf3D70087/N9. (A) Parasite reduction rates from day 3 to day 7 compared to control (mean values, *n* = 5). (B) Five mice (M1 ■, M2 ▲, M3 ⧫, M4 •, M5 ×) were treated with a 12 mg/kg single oral dose of M5717 at day 3 postinfection (red arrow). (C) Five mice (M1’ ■, M2’ ▲, M3’ ⧫, M4’ •, M5’ ×) were treated with a single oral dose of 12 mg/kg M5717 and 6 mg/kg pyronaridine at day 3 postinfection (red arrow). (D) Five mice (M1” ■, M2” ▲, M3” ⧫, M4” •, M5” ×) were treated with a 12 mg/kg single oral dose of M5717 and pyronaridine at day 3 postinfection (red arrow). Parasitemia was measured by microscopy. Gray areas correspond to retreatments with the same initial dosing regimen at day 3 postinfection.

To further explore this combination and to bring more relevance, we decided to dose three groups of five mice. All groups were treated with the same suboptimal dose of M5717, i.e., 12 mg/kg single oral dose. We then added pyronaridine in two groups with 6 mg/kg and 12 mg/kg single oral doses, respectively. As previously described, a single oral dose of 12 mg/kg of M5717 displayed a delayed parasite clearance (Fig. 2A), where the lowest limit of quantification (LLQ) was reached after day 7 (i.e., day 8) postinfection (7). When combining M5717 with 6 mg/kg of pyronaridine, a similar parasite clearance time was observed, suggesting that the latter dose was suboptimal. However, on increasing the dose of pyronaridine to 12 mg/kg, the parasite clearance time was substantially increased and reached the LLQ at day 6 (Fig. 2A).

With a single 12 mg/kg oral dose of M5717, all mice displayed parasite recrudescence above the LLQ (≥0.01%) around the third week postinfection (Fig. 2B). Mice with early recrudescence (D17, D18, and D19) were not susceptible to retreatment with the same dosing regimen, as opposed to mice with later-recrudescing parasites (D23 and D24), which responded partially to retreatment.

With the same dose of M5717 (12 mg/kg), in combination with a suboptimal dose of pyronaridine at 6 mg/kg, the recrudescence pattern was partially affected (Fig. 2C). Mice had parasites reemerging between days 21 and 33 with a full response (i.e., similar rate of kill as in the initial treatment), from which two recrudesced again 28 to 36 days after retreatment, suggesting that the overall dose was suboptimal. When the dose of pyronaridine was increased to a more optimal dose of 12 mg/kg, the recrudescence pattern was altered again, with three mice showing no recrudescence at all by day 80 (Fig. 2D). The two mice with late recrudescence at around 7 weeks postinfection responded fully to the same retreatment.

### *In vivo* selection of mutants.

**FIG 3:**
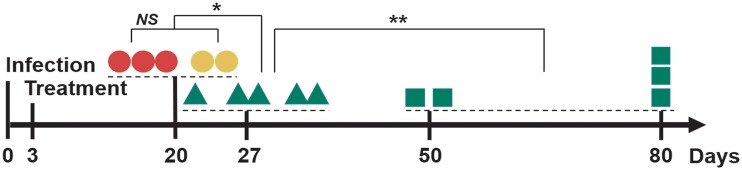
Time to recrudescence (≥0.01% parasitemia) and parasite mutation profiles as a function of single oral treatments with 12 mg/kg single oral doses of M5717 (•), 12 mg/kg M5717 and 6 mg/kg pyronaridine (▲), and 12 mg/kg single oral dose of M5717 and pyronaridine (■) at day 3 postinfection. Parasitemia was measured by microscopy. Red icons correspond to M5717-mutant parasites with a high grade of resistance. Orange icons correspond to M5717-mutant parasites with a medium grade of resistance. Green icons correspond to wild-type parasites. *, *P* < 0.05; **, *P* < 0.01; NS, not significant.

We observed that monotherapy with M5717 in the murine P. falciparum SCID model can lead to selection of mutant parasites, and the addition of a suboptimal dose of pyronaridine during a combination treatment can clear M5717-related resistance (Fig. 3; see Materials and Methods).

In the 12 mg/kg M5717 treatment arm, parasites reemerged in all mice. The median time to recrudescence of parasites in mice (M1, M2, M3, *n* = 3) showing no response to retreatment was 18 days. Sequencing indicated the mutation Y186N (Table S3) in the P. falciparum *eEF2* site that has conferred high-grade resistance (>3,000-fold shift of half maximal inhibitory concentration [IC_50_]) in prior *in vitro* studies (7). Mice exhibiting later recrudescence (M4 and M5, *n* = 2) showed a median recrudescence at 23.5 days and partially responded to retreatment. Sequencing of these mice showed the mutation T753N in the P. falciparum *eEF2* site. Nearby mutations P754A, P754S, and L755F had IC_50_ fold shifts of 135-, 200-, and 660-fold, respectively, in prior *in vitro* studies, with the position 754 mutations corresponding to medium-grade resistance (7). Although the difference between early and late recrudescence groups was not significant (*P* = 0.06), the comparison was based on very small sample sizes (*n* = 3 and *n* = 2), so the *P* value of borderline significance should not be interpreted as no difference in recrudescence time. Overall, resistance evolved in 5/5 mice in the 12 mg/kg M5717 arm, with a median time to recrudescence of 19 days (Table S5).

When adding a suboptimal dose of pyronaridine of 6 mg/kg to the 12 mg/kg of M5717 (*n* = 5), the median time to recrudescence was significantly shifted to 26 days (*P* = 0.0127). All parasites were sequenced and were found to be wild type, confirming that (i) combination with pyronaridine effectively suppressed the emergence of M5717-resistant parasites and (ii) the combined doses were subtherapeutic. Finally, when increasing the dose of pyronaridine to 12 mg/kg with 12 mg/kg of M5717 (*n* = 5), 3 mice were deemed cured, i.e., no recrudescence at day 80, while 2 mice (M1” and M2”) showed a median recrudescence on day 50 with wild-type parasites, as determined by sequencing. This increase in dose of pyronaridine significantly shifted the outcome compared to a suboptimal dose of pyronaridine (*P* = 0.0018).

## DISCUSSION

The pressing need for new antimalarial combinations is mandated by the current decline in efficacy in artemisinin-based combination therapies in Southeast Asia, which has historically served as the bellwether for resistance emerging or spreading globally. Here, we report PK/PD studies on combinations of two potent antimalarials, M5717 and pyronaridine. Our study employed *in vitro* cultures of asexual blood-stage P. falciparum parasites and *in vivo* trials using the P. falciparum SCID mouse model. Overall the parasitological behavior was additive, and no pharmacokinetic interactions were observed when the two substances were given in combination.

We hypothesized that M5717, the slower-acting partner that inhibits the parasite eukaryotic elongation factor *PfeEF2*, and pyronaridine, the faster-acting partner that inhibits the formation of hemozoin, could be an interesting pairing of antimalarials given their potential matching pharmacokinetic profiles (e.g., similar t_1/2_) and complementary pharmacodynamic characteristics. Pyronaridine was recently approved by the European Medicines Agency (EMA) (Article 58) in combination with artesunate under the brand name Pyramax by Shin-Poong Pharmaceutical, and several reports have studied the *in vitro* combination of pyronaridine and artesunate (19, 23, 24). This prompted a first *in vitro* assessment entailing isobologram analyses after various durations of exposure. Results were consistent with a mostly additive effect (Table 1).

Given their additive effect and long t_1/2_ profiles in mice, namely, 37 h for M5717 and 74 h for pyronaridine, we proceeded with single oral dosing in the P. falciparum SCID mice model. For both compounds we measured pharmacokinetic (AUC_0-inf_) and pharmacodynamic (% parasitemia, day of recrudescence, sequencing) parameters. No pharmacokinetic interactions were observed between the two substances in the peripheral blood (Table 2). Overall, the two compounds showed additive parasitological properties, i.e., the rate of kill of pyronaridine seemed unaffected at therapeutic doses, and a delay in recrudescence was observed in combined treatment compared to monotherapy. Alone, pyronaridine was able to linearly extend the recrudescence time with doses of 6, 12, or 36 mg/kg. Conversely, a plateau to recrudescence was reached from 12 mg/kg to 30 mg/kg with M5717.

DNA sequencing showed that M5717-resistant parasites had been selected, i.e., mutation occurred in the *eEF2* locus that conferred a high degree of resistance. As it has been shown for atovaquone in the P. berghei mouse model, in-host selection of mutant parasites is possible and can be dose dependent, i.e., high doses yield highly resistant mutant selection whereas low doses can show selection of low-grade resistance (25, 26).

To further investigate the effects of the combination on parasite recrudescence and M5717-resistant mutant selection, we chose a suboptimal dose of M5717 fixed at 12 mg/kg in all of the three treatment arms (*n* = 5 for each) followed by an increasing dose of the partner agent, pyronaridine at 6 or 12 mg/kg (Fig. 2). Monotherapy with M5717 at 12 mg/kg led to median recrudescence at day 20 with two subpopulations of mutants. By sequencing the monotherapy arm parasites, we showed that mutation in the *eEF2* locus could be translated into no or partial response to retreatment with M5717 (Fig. 2B). Next, by gradually increasing the dose of the partner drug pyronaridine to a therapeutically relevant level, the parasite recrudescence pattern was favorably altered. We found that the addition of a suboptimal dose of pyronaridine totally suppressed the emergence of mutant parasites and also postponed the time to recrudescence significantly compared to M5717 alone (*P* < 0.05). In addition, increasing the dose of pyronaridine to 12 mg/kg along with a 12 mg/kg dose of M5717 gave a cure rate of 60% at day 80 and significantly postponed the day of recrudescence for the other mice (*P* < 0.001). These findings indicate that M5717 should not be exposed to parasites as a monotherapy and provide evidence that the constant presence of a partner drug in a matching pharmacokinetic and pharmacodynamic fashion is recommended, assuming that the observations made in mice are translatable to humans.

The results described are the first report suggesting that M5717, a *Plasmodium eEF2* inhibitor, and pyronaridine, a hemozoin formation inhibitor, could be an effective, long-lasting antimalarial combination with complementary pharmacodynamic profiles. The combination maintains fast clearance of blood-stage parasites while adding exoerythrocytic activity, consequently providing additional benefits for treatment, such as prophylaxis or transmission-blocking potential. Our isobologram data, combined with drug combination experiments in the P. falciparum SCID mouse model coupled with pharmacokinetic sampling and parasite sequencing, highlights the promise of this potential new antimalarial combination.

Overall, molecules with a high propensity for generating resistance should be combined with a partner drug having, at a minimum, a matching anti-parasitic exposure with no or low propensity to generating resistance at relevant parasite inocula. If, in addition, these molecules have activity on exoerythrocytic stages (liver and gametes), then targeting populations with low parasitemia for prevention and prophylaxis rather than cure could leverage their full potential in view of supporting the malaria-elimination agenda.

## MATERIALS AND METHODS

### *In vitro* isobolograms.

IC_50_ and drug interaction studies were performed as previously described (27, 28). Drug solutions were diluted with hypoxanthine-free culture medium to initial concentrations of 10 times the predetermined IC_50_ (named S10× IC_50_). The S10× IC_50_ solutions for each compound were combined in ratios of 1 + 3, 1 + 1, or 3 + 1, and each drug was also tested alone (Table 1). Aliquots of 100 μl of single and combination drug solutions were then introduced into 96-well plates to give duplicate rows and drugs were diluted 2-fold across a range of 10 concentrations. Equal volumes of parasite culture with a parasitemia of 0.3% in a 2.5% erythrocyte suspension were added and the test plates were incubated for 24 h, 48 h, or 72 h. Parasite growth was measured by the incorporation of radiolabeled [^3^H]hypoxanthine (0.25 μCi in a volume of 50 μl hypoxanthine-free culture medium) added 8 h (for 24 h assay duration) or 24 h (48 h and 72 h assay duration) prior to the termination of the test. Cultures were harvested onto glass fiber filters, washed, and the radioactivity was counted using a MicroBetaplate liquid scintillation counter and recorded as counts per minute (cpm) per well. Results are expressed as a percentage of the untreated controls (see the supplemental material for more details).

Isobolograms were constructed by plotting the fractional inhibitory concentrations producing half-maximal growth (FIC_50_s) of drug A against the FIC_50_s of drug B for each of the three drug ratios. A concave curve indicated synergy, a straight line indicated additivity, and a convex curve indicated antagonism (see supplemental material). To obtain numeric values for the interaction, results were expressed as the sum of the FIC_50A_ and FIC_50B_. Sum FIC_50_ values characterize the interactions as follows: synergism when the ΣFIC_50_ is ≤0.5; antagonism when the ΣFIC_50_ is >2.0; no interaction when 0.5 < ƩFIC_50_ ≤2.0 (29).

### *In vivo* SCID mouse model.

Compound efficacy was assessed in the murine P. falciparum SCID model essentially as described by Jimenez-Díaz et al. (20). Briefly, alone or in combination, M5717 (succinate salt by Merck KGaA [MSC2576186-B4]) and pyronaridine (tetraphosphate salt, Sigma-Aldrich [P0049]) were formulated in 7% Tween80, 3% ethanol and administered to a cohort of age-matched female immunodeficient NOD-*scid IL-2Rγ^null^* mice (Jackson Laboratory, Bar Harbor, ME). These mice had been previously engrafted with human erythrocytes (generously provided by the blood bank in Zürich, Switzerland). Prior to compound treatment, mice were intravenously infected with 2 × 10^7^ P. falciparum Pf3D7^0087/N9^ -infected erythrocytes (day 0) (30). On day 3 after infection, mice, in groups of 2 to 5, were randomly allocated to treatments that were administered with a single oral gavage at 10 ml/kg (Table 2 and Fig. 2). Parasitemia was measured by microscopy. Chimerism was monitored by flow cytometry using an anti-murine erythrocyte TER119 monoclonal antibody (Pharmingen, San Diego, CA) and SYTO-16 and then analyzed by flow cytometry in serial blood samples (2 μl) collected every 2 to 3 days until the completion of the experiment (Table 2, day 60 and Fig. 2, day 80). Parasite recrudescence was detected by microscopy. Once parasitemia exceeded 0.5%, then 100 to 500 μl of blood was collected and stored at –80°C for subsequent sequence analysis. Animals received a second treatment with the same dose and if applicable the same combination of compounds as previously administered in that dose group (Fig. 2). Efficacy was again monitored by microscopy. If recrudescence occurred again then new blood samples were stored at –80°C.

*In vivo* studies conducted at the Swiss TPH, Basel, were approved by the veterinary authorities of the Canton Basel-Stadt (permit no. 2303) based on Swiss cantonal (Verordnung Veterinäramt Basel-Stadt) and national regulations (the Swiss animal protection law, Tierschutzgesetz).

### Pharmacokinetic analysis.

The test compounds’ concentrations in blood were assessed in order to determine pharmacokinetic parameters in mice from the efficacy study. Peripheral blood samples (20 μl) were sampled at different time points (1, 2, 4, 6, 24, 48, 72, 96, 168, and 216 h post treatment), mixed with 20 μl of H_2_O Milli Q and immediately frozen on dry ice. Samples were stored at –80°C until analysis. Blood from control mice was used for bioanalysis calibration and QC purposes. For liquid chromatography with tandem mass spectrometry (LC-MS/MS) analysis, the frozen samples were thawed and treated with two volumes equivalent of acetonitrile containing the internal standard. After centrifugation, one volume equivalent of supernatant was diluted with one volume equivalent of water containing heptafluorobutyric acid (HFBA). The extracts were analyzed by LC-MS/MS (quantification by HESI ionization in positive ion mode) performed at Swiss BioQuant AG (Switzerland). A noncompartmental analysis was performed to determine pharmacokinetic parameters using the Phoenix WinNonlin program (version 6.3).

### Sequencing P. falciparum blood samples for *eEF2* mutations.

Blood samples collected from mice with recrudescent infections were lysed in 0.1% saponin and washed twice with 1× phosphate-buffered saline (PBS), with repeat rounds as necessary. DNA was then extracted using the DNeasy blood and tissue kit (Qiagen). The 2.7 kb *PfeEF2* gene was PCR-amplified using flanking primers (Table S2). The PCR conditions for the initial amplification were: 95°C for 3 min, 45 rounds of 98°C for 20 s, 55°C for 30 s, and 68°C for 2.5 min, with a final extension of 3 min at 68°C. Agarose gel (1%) electrophoresis was used to confirm the PCR product size. Sanger sequencing was carried out by Genewiz Inc. In addition to the amplification primers, nine additional sequencing primers were used to fully sequence the *PfeEF2* gene (Table S2). All the sequences were aligned to the wild-type *eEF2* 3D7 sequence and analyzed on DNASTAR SeqMan Pro 15 software. Electropherograms were visually inspected to identify mixed sequences indicating multiple subpopulations.

### Statistical analysis.

Time-to-recrudescence data were analyzed using the Kaplan-Meier method. Log-rank test was performed for two-group comparisons. Kaplan-Meier curves and log-rank test *P* values, as well as median estimates for time to recrudescence, are presented (SAS, Version 9.4. Cary, NC: SAS Institute Inc.; 2014).

## Supplementary Material

Supplemental file 1

## References

[1] Blasco B, Leroy D, Fidock DA 2017 Antimalarial drug resistance: linking *Plasmodium falciparum* parasite biology to the clinic. Nat Med 23:917–928. doi:10.1038/nm.4381. 28777791 PMC5747363

[2] World Health Organization. 2018 Artemisinin resistance and artemisinin-based combination therapy efficacy. World Health Organization, Geneva, Switzerland https://apps.who.int/iris/handle/10665/274362.

[3] van der Pluijm RW, Imwong M, Chau NH, Hoa NT, Thuy-Nhien NT, Thanh NV, Jittamala P, Hanboonkunupakarn B, Chutasmit K, Saelow C, Runjarern R, Kaewmok W, Tripura R, Peto TJ, Yok S, Suon S, Sreng S, Mao S, Oun S, Yen S, Amaratunga C, Lek D, Huy R, Dhorda M, Chotivanich K, Ashley EA, Mukaka M, Waithira N, Cheah PY, Maude RJ, Amato R, Pearson RD, Gonçalves S, Jacob CG, Hamilton WL, Fairhurst RM, Tarning J, Winterberg M, Kwiatkowski DP, Pukrittayakamee S, Hien TT, Day NP, Miotto O, White NJ, Dondorp AM 2019 Determinants of dihydroartemisinin-piperaquine treatment failure in *Plasmodium falciparum* malaria in Cambodia, Thailand, and Vietnam: a prospective clinical, pharmacological, and genetic study. Lancet Infect Dis 19:952–961. doi:10.1016/S1473-3099(19)30391-3. 31345710 PMC6715822

[4] White NJ, Olliaro PL 1996 Strategies for the prevention of antimalarial drug resistance Rationale for combination chemotherapy for malaria. Parasitol Today 12:399–401. doi:10.1016/0169-4758(96)10055-7. 15275291

[5] Ding XC, Ubben D, Wells T 2012 A framework for assessing the risk of resistance for anti-malarials in development. Malar J 11:292. doi:10.1186/1475-2875-11-292. 22913649 PMC3478971

[6] Cowell AN, Winzeler EA 2019 The genomic architecture of antimalarial drug resistance. Brief Funct Genomics 18:314–328. doi:10.1093/bfgp/elz008. 31119263 PMC6859814

[7] Baragaña B, Hallyburton I, Lee MCS, Norcross NR, Grimaldi R, Otto TD, Proto WR, Blagborough AM, Meister S, Wirjanata G, Ruecker A, Upton LM, Abraham TS, Almeida MJ, Pradhan A, Porzelle A, Luksch T, Martínez MS, Luksch T, Bolscher JM, Woodland A, Norval S, Zuccotto F, Thomas J, Simeons F, Stojanovski L, Osuna-Cabello M, Brock PM, Churcher TS, Sala KA, Zakutansky SE, Jiménez-Díaz MB, Sanz LM, Riley J, Basak R, Campbell M, Avery VM, Sauerwein RW, Dechering KJ, Noviyanti R, Campo B, Frearson JA, Angulo-Barturen I, Ferrer-Bazaga S, Gamo FJ, Wyatt PG, Leroy D, Siegl P, Delves MJ, Kyle DE, Wittlin S, Marfurt J, Price RN, Sinden RE, Winzeler EA, Charman SA, Bebrevska L, Gray DW, Campbell S, Fairlamb AH, Willis PA, Rayner JC, Fidock DA, Read KD, Gilbert IH 2015 A novel multiple-stage antimalarial agent that inhibits protein synthesis. Nature 522:315–320. doi:10.1038/nature14451. 26085270 PMC4700930

[8] Baragaña B, Norcross NR, Wilson C, Porzelle A, Hallyburton I, Grimaldi R, Osuna-Cabello M, Norval S, Riley J, Stojanovski L, Simeons FR, Wyatt PG, Delves MJ, Meister S, Duffy S, Avery VM, Winzeler EA, Sinden RE, Wittlin S, Frearson JA, Gray DW, Fairlamb AH, Waterson D, Campbell SF, Willis P, Read KD, Gilbert IH 2016 Discovery of a quinoline-4-carboxamide derivative with a novel mechanism of action, multistage antimalarial activity, and potent *in vivo* efficacy. J Med Chem 59:9672–9685. doi:10.1021/acs.jmedchem.6b00723. 27631715 PMC5108032

[9] Arez F, Rebelo S, Fontinha D, Simão D, Martins T, Machado M, Fischli C, Oeuvray C, Badolo L, Carrondo M, Rottmann M, Spangenberg T, Brito C, Greco B, Prudêncio M, Alves PM 2019 Flexible 3D cell-based platforms for the discovery and profiling of novel drugs targeting *Plasmodium* hepatic infection. ACS Infect Dis 5:1831–1842. doi:10.1021/acsinfecdis.9b00144. 31479238

[10] Zheng XY, Xia Y, Gao FH, Chen C 1979 Synthesis of 7351, a new antimalarial drug (author’s transl). Yao Xue Xue Bao 14:736–737. 554435

[11] Zheng XY, Chen C, Gao FH, Zhu PE, Guo HZ 1982 Synthesis of new antimalarial drug pyronaridine and its analogues (author’s transl). Yao Xue Xue Bao 17:118–125. 7102320

[12] Feng Z, Wu ZF, Wang CY, Jiang NX 1987 Pharmacokinetics of pyronaridine in malaria patients. Zhongguo Yao Li Xue Bao 8:543–546. 3330403

[13] Chang C, Lin-Hua T, Jantanavivat C 1992 Studies on a new antimalarial compound: pyronaridine. Trans R Soc Trop Med Hyg 86:7–10. doi:10.1016/0035-9203(92)90414-8. 1566313

[14] Croft SL, Duparc S, Arbe-Barnes SJ, Craft JC, Shin C-S, Fleckenstein L, Borghini-Fuhrer I, Rim H-J 2012 Review of pyronaridine anti-malarial properties and product characteristics. Malar J 11:270. doi:10.1186/1475-2875-11-270. 22877082 PMC3483207

[15] Chang C 2018 Artemisinin-based and other antimalarials: detailed account of studies by Chinese scientists who discovered and developed them, p 571–607. Academic Press, New York, NY.

[16] Jayaraman SD, Ismail S, Nair NK, Navaratnam V 1997 Determination of pyronaridine in blood plasma by high-performance liquid chromatography for application in clinical pharmacological studies. J Chromatogr B Biomed Sci Appl 690:253–257. doi:10.1016/s0378-4347(96)00410-0. 9106050

[17] Sanz LM, Crespo B, De-Cózar C, Ding XC, Llergo JL, Burrows JN, García-Bustos JF, Gamo F-J 2012 *P falciparum in vitro* killing rates allow to discriminate between different antimalarial mode-of-action. PLoS One 7:e30949. doi:10.1371/journal.pone.0030949. 22383983 PMC3285618

[18] Auparakkitanon S, Chapoomram S, Kuaha K, Chirachariyavej T, Wilairat P 2006 Targeting of hematin by the antimalarial pyronaridine. Antimicrob Agents Chemother 50:2197–2200. doi:10.1128/AAC.00119-06. 16723583 PMC1479140

[19] Henrich PP, O'Brien C, Sáenz FE, Cremers S, Kyle DE, Fidock DA 2014 Evidence for pyronaridine as a highly effective partner drug for treatment of artemisinin-resistant malaria in a rodent model. Antimicrob Agents Chemother 58:183–195. doi:10.1128/AAC.01466-13. 24145526 PMC3910733

[20] Jiménez-Díaz MB, Mulet T, Viera S, Gómez V, Garuti H, Ibáñez J, Alvarez-Doval A, Shultz LD, Martínez A, Gargallo-Viola D, Angulo-Barturen I 2009 Improved murine model of malaria using *Plasmodium falciparum* competent strains and non-myelodepleted NOD-scid IL2Rgammanull mice engrafted with human erythrocytes. Antimicrob Agents Chemother 53:4533–4536. doi:10.1128/AAC.00519-09. 19596869 PMC2764183

[21] Childs GE, Hausler B, Milhous W, Chen C, Wimonwattrawatee T, Pooyindee N, Boudreau EF 1988 *In vitro* activity of pyronaridine against field isolates and reference clones of *Plasmodium falciparum*. Am J Trop Med Hyg 38:24–29. doi:10.4269/ajtmh.1988.38.24. 3277460

[22] Snyder C, Chollet J, Santo-Tomas J, Scheurer C, Wittlin S 2007 *In vitro* and *in vivo* interaction of synthetic peroxide RBx11160 (OZ277) with piperaquine in *Plasmodium* models. Exp Parasitol 115:296–300. doi:10.1016/j.exppara.2006.09.016. 17087929

[23] Vivas L, Rattray L, Stewart L, Bongard E, Robinson B, Peters W, Croft SL 2008 Anti-malarial efficacy of pyronaridine and artesunate in combination *in vitro* and *in vivo*. Acta Trop 105:222–228. doi:10.1016/j.actatropica.2007.12.005. 18279817

[24] Ringwald P, Eboumbou EC, Bickii J, Basco LK 1999 *In vitro* activities of pyronaridine, alone and in combination with other antimalarial drugs, against *Plasmodium falciparum*. Antimicrob Agents Chemother 43:1525–1527. doi:10.1128/AAC.43.6.1525. 10348789 PMC89315

[25] Nuralitha S, Murdiyarso LS, Siregar JE, Syafruddin D, Roelands J, Verhoef J, Hoepelman AIM, Marzuki S 2017 Within-host selection of drug resistance in a mouse model reveals dose-dependent selection of atovaquone resistance mutations. Antimicrob Agents Chemother 61:e01867-16. doi:10.1128/AAC.01867-16. 28193656 PMC5404568

[26] Stickles AM, Smilkstein MJ, Morrisey JM, Li Y, Forquer IP, Kelly JX, Pou S, Winter RW, Nilsen A, Vaidya AB, Riscoe MK 2016 Atovaquone and ELQ-300 combination therapy as a novel dual-site cytochrome bc1 inhibition strategy for malaria. Antimicrob Agents Chemother 60:4853–4859. doi:10.1128/AAC.00791-16. 27270285 PMC4958223

[27] Canfield CJ, Pudney M, Gutteridge WE 1995 Interactions of atovaquone with other antimalarial drugs against *Plasmodium falciparum in vitro*. Exp Parasitol 80:373–381. doi:10.1006/expr.1995.1049. 7729473

[28] Fivelman QL, Adagu IS, Warhurst DC 2004 Fixed-ratio isobologram method for studying *in vitro* interactions between atovaquone and proguanil or dihydroartemisinin against drug-resistant strains of *Plasmodium falciparum*. Antimicrob Agents Chemother 48:4097–4102. doi:10.1128/AAC.48.11.4097-4102.2004. 15504827 PMC525430

[29] Odds FC 2003 Synergy, antagonism and what the chequerboard puts between them. J Antimicrob Chemother 52:1. doi:10.1093/jac/dkg301. 12805255

[30] Angulo-Barturen I, Jiménez-Díaz MB, Mulet T, Rullas J, Herreros E, Ferrer S, Jiménez E, Mendoza A, Regadera J, Rosenthal PJ, Bathurst I, Pompliano DL, Gómez de las Heras F, Gargallo-Viola D 2008 A murine model of falciparum-malaria by *in vivo* selection of competent strains in non-myelodepleted mice engrafted with human erythrocytes. PLoS One 3:e2252. doi:10.1371/journal.pone.0002252. 18493601 PMC2375113

