# Supplementary material for: Preclinical Antimalarial Combination Study of M5717, a Plasmodium falciparum Elongation Factor 2 Inhibitor, and Pyronaridine, a Hemozoin Formation Inhibitor
Source: Antimicrob Agents Chemother. 2020 Mar 24;64(4):e02181-19. doi: 10.1128/AAC.02181-19 (PMC7179297; doi:10.1128/AAC.02181-19)
Supplement: Supplemental file 1 [file AAC.02181-19-s0001.pdf]

SUPPLEMENTAL MATERIAL

Preclinical Antimalarial Combination Studies: The Case of M5717, a *P. falciparum* Elongation Factor 2 Inhibitor and Pyronaridine, a Hemozoin Formation Inhibitor

Matthias Rottmann[1,2], Brian Jonat[3], Christin Gumpp[1,2], Satish K. Dhingra[4], Marla J. Giddins[4], Xiaoyan Yin[5], Lassina Badolo[6], Beatrice Greco[7], David A. Fidock[4,8], Claude Oeuvray[7], Thomas Spangenberg[7]*

1 Department of Medical Parasitology and Infection Biology, Swiss Tropical and Public Health Institute, Socinstr. 57, CH-4051 Basel, Switzerland

2 University of Basel, Basel, Switzerland

3 Department of Pediatrics, Columbia University Irving Medical Center, New York, NY 10032 USA

4 Department of Microbiology and Immunology, Columbia University Irving Medical Center, New York, NY 10032 USA

5 Global Statistics for NDD, Immunology, Endocrinology, Fertility & Others, EMD Serono, Billerica MA, USA

6 Discovery and Development Technologies, Merck Healthcare KGaA, Frankfurter Strasse 250, 64293 Darmstadt, Germany

7 Global Health Institute of Merck, Ares Trading S.A., Route de Crassier 1, 1262 Eysins, Switzerland, a subsidiary of Merck KGaA, Darmstadt, Germany

8 Division of Infectious Diseases, Department of Medicine, Columbia University Irving Medical Center, New York, NY 10032 USA

*Corresponding author: thomas.spangenberg@merckgroup.com

***In vitro* isobolograms**

Compounds were tested against intraerythrocytic forms of *P. falciparum* derived from asynchronous stock cultures of lab strain NF54 (Schiphol airport strain of unknown origin), as previously described.[i,ii] The culture medium was a variation of that previously described[iii,iv] consisting of RPMI 1640 supplemented with 0.5% ALBUMAX® II, 25 mM HEPES, 25 mM $NaHCO_3$ (pH 7.3), 0.36 mM hypoxanthine and 100 µg/mL neomycin. Human type A^+ erythrocytes served as host cells. The cultures were kept at 37°C in an atmosphere of 3% O_2, 4% CO_2 and 93% N_2 in humidified modular chambers. Testing of the individual drugs was carried out in 96-well microtiter plates. The compounds were dissolved in DMSO (10 mg/mL), prediluted in hypoxanthine-free culture medium and titrated in 100 µL duplicates over a 64-fold range. After addition of an equal volume of parasite culture with a parasitemia of 0.3% in a 2.5% erythrocyte suspension, the test plates were incubated under the conditions described above for 24 h, 48 h or 72 h. Parasite growth was measured by the incorporation of radiolabelled [^{3}H]hypoxanthine (0.25 µCi in a volume of 50 µL hypoxanthine-free culture medium) added 8 h (for 24 h assay duration) or 24 h (48 h and 72 h assay duration) prior to the termination of the test. Cultures were harvested onto glass-fiber filters and washed with distilled water. The radioactivity was counted using a MicroBetaplate liquid scintillation counter (Wallac, Zürich, Switzerland) and the results recorded as counts per minute (cpm) per well at each drug concentration and expressed as percentage of the untreated controls. Fifty percent inhibitory concentrations (IC_{50}) were determined by linear interpolation.[v]

For data interpretation, the IC_{50}s of the drugs in combination were expressed as fractions of the IC_{50}s of the drugs alone. These fractions were called “Fractional Inhibitory Concentrations” (FIC) for drug A and for drug B, respectively.

$$\mathbf{FIC\ Drug\ A} = \frac{\mathbf{IC50\ A\ (B)}}{\mathbf{IC50\ A}}$$

IC_{50} A (B): 50% inhibitory concentration of drug A in presence of drug B

IC_{50} A: 50% inhibitory concentration of drug A alone

Formulation

Pyronaridine (tetraphosphate salt) and M5717 (succinate salt) were solubilized in a vehicle consisting of 70% Tween-80 and 30% ethanol, followed by a 10-fold dilution in H_2O. All preparations resulted in a yellow, liquid and clear solution.

Pharmacokinetic parameters

The pharmacokinetic analysis was performed non-compartmentally using the extravascular model. For the analysis, concentrations below the LLOQ were set to 0. The following pharmacokinetic parameters of the analytes were derived from the concentration-time profiles.

C_0	The pre-dose concentration assumed to be 0 on the day of oral dosing.
t_{max}	Time where the concentration in matrix is the highest.
C_{max}	Peak concentration.
C_{max}/dose	C_{max} normalized to the dose of 1 mg/kg.
$AUC_{0\text{-}t}$	Area under the matrix concentration-time curve from 0 h to time t calculated using the linear trapezoidal rule, using C_0 as defined above.
$AUC_{0\text{-}\infty}$	Area under the matrix concentration-time curve from 0 h to infinity calculated using the linear trapezoidal rule, using C_0 as defined above.
$AUC_{0\text{-}\infty}$/dose	$AUC_{0\text{-}\infty}$ normalized to a dose of 1 mg/kg.

Software for calculations: Phoenix WinNonlin program (version 6.3).

All concentration values and pharmacokinetic parameters were rounded to 3 significant digits. The numbers listed for %bias and %CV were rounded to 1 decimal place.

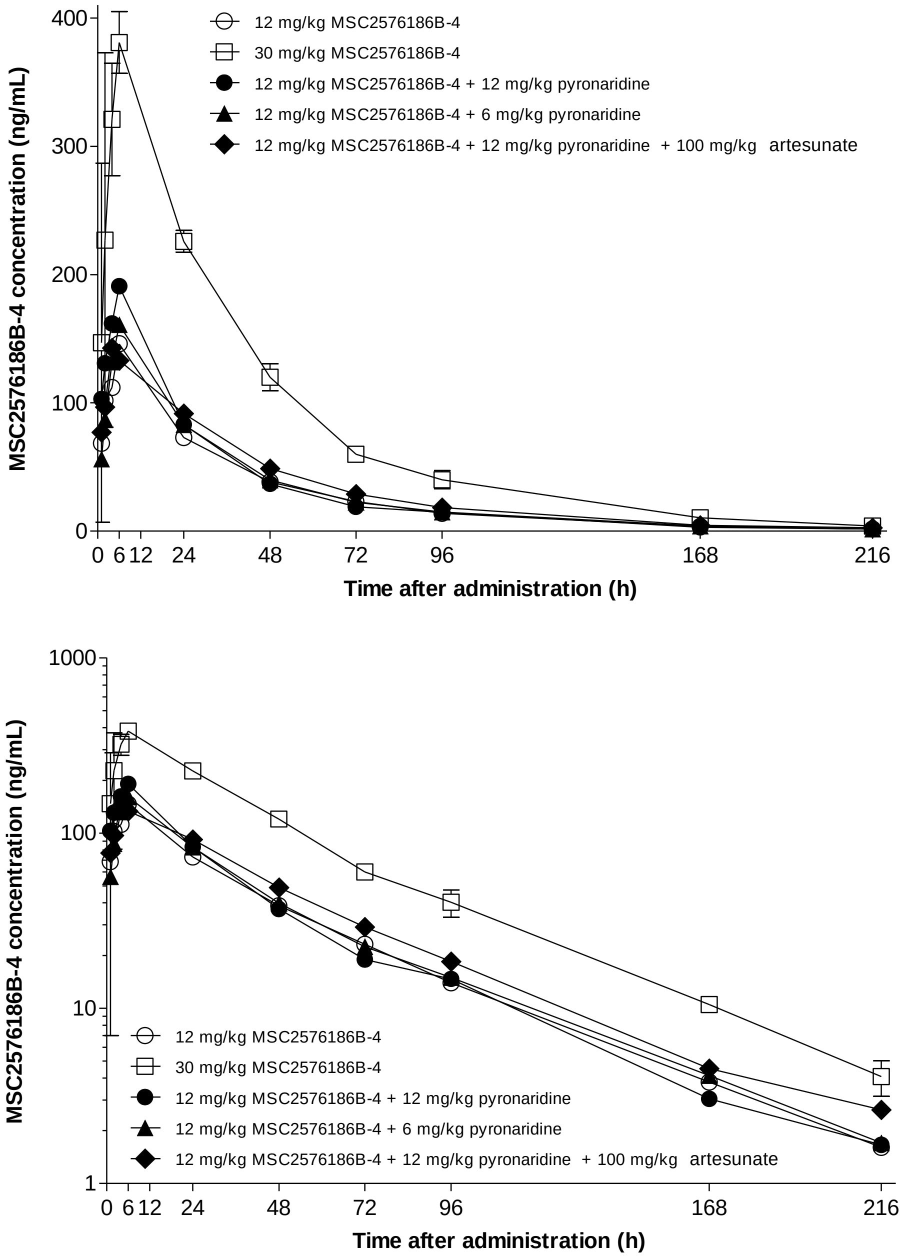

Figure S1. Mean (N=2-3) MSC2576186B-4 (M5717) blood concentration profiles after single-dose oral administration alone or in combination with pyronaridine and artesunate in mice (linear and semi-log scales)

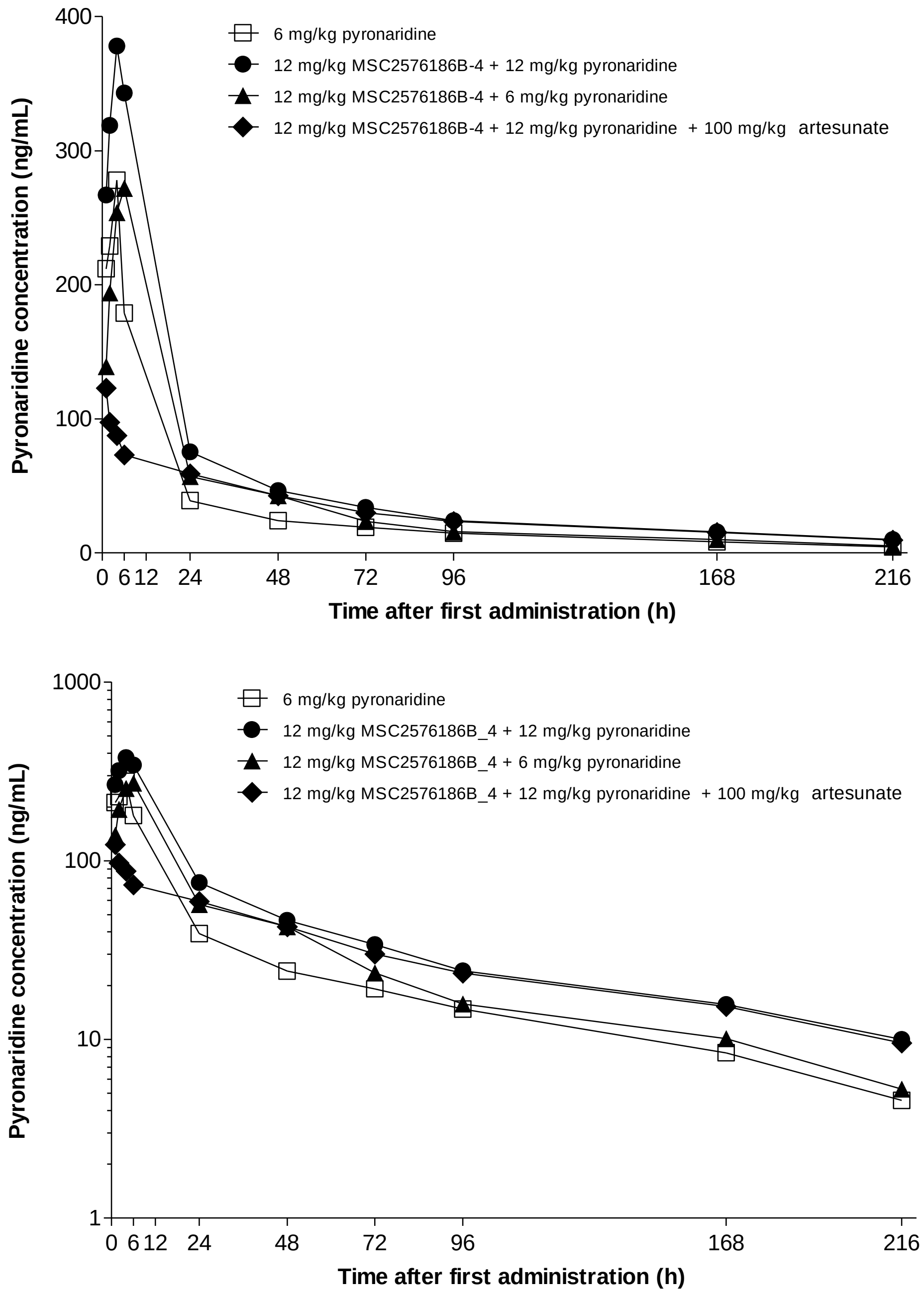

Figure S2. Mean (N=2-3) pyronaridine blood concentration profiles after single-dose oral administration alone or in combination with MSC2576186B-4 (M5717) and artesunate in mice (linear and semi-log scales).

Table S1: **Summary of parasite recrudescence, mutations and response to treatment after administration of M5717 as monotherapy or in combination with Pyronaridine**. Mice in each treatment group were ranked by day of recrudescence. Day of recrudescence was defined as the day when parasitemia was again detectable after falling below the lowest limit of quantification (LLQ). Full response to retreatment was defined as parasitemia falling below LLQ after drug administration. Partial response was defined as a drop in parasitemia after treatment, but without achieving LLQ. No response was defined as no drop in parasitemia after treatment. Color coding refers to degree of resistance conferred by sequenced mutations: red indicating high grade, yellow indicating medium, and green indicating wild type.

Day of recrudescence, mutations & drug response								
Single Oral Dose		M1, M1', M1"		M2, M2', M2"	M3, M3', M3"	M4, M4', M4"	M5, M5', M5"	
**M5717**	**PYRO**							
**12 mg/kg**	-	**D17**		**D18**	**D19**	**D23**	**D24**	
Response to retreatment		***No***		***No***	***No***	***Partial***	***Partial***	
Mutation, AA position		***Yes, 186***		***Yes, 186***	***Yes, 753***	***Yes, 753***	***Yes, 186***	
**12 mg/kg**	**6 mg/kg**	**D21**	**D49**	**D25**	**D26**	**D32**	**D33**	**D69**
Response to retreatment		***Full***	***Full***	***Full***	***Full***	***Full***	***Full***	***ND***
Mutation, AA position		***WT***	***WT***	***WT***	***WT***	***WT***	***WT***	
**12 mg/kg**	**12 mg/kg**	**D47**		**D52**	***No relapse***	***No relapse***	***No relapse***	
Response to retreatment		***Full***		***Full***	***NA***	***NA***	***NA***	
Mutation, AA position		***WT***		***WT***	***WT***	***WT***	***WT***	

P. falciparum **eEF2 mutation sites**

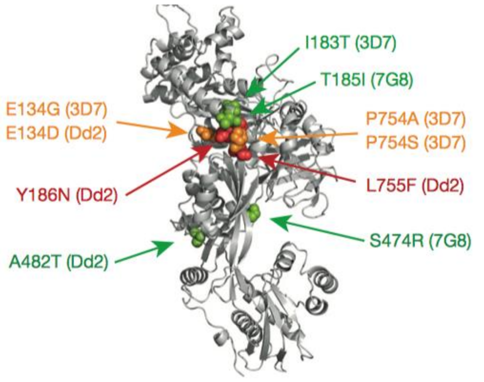

From Baragaña B, Hallyburton I, Lee MCS, Norcross NR, Grimaldi R, Otto T et al. A novel multiple-stage antimalarial agent that inhibits protein synthesis. Nature 2015; 522: 315−320.

Table S2: Primers used for *PfeEF2* PCR amplification and sequencing.

Primer Name	Sequence (5'→ 3')	PCR Function
**p7580**	**CTTTACGGTAGATCAAGTTCGTG**	***Outer Flank, Sequencing***
**p7582**	**AATATTACCGTGTATTTGTATGTAC**	***Outer Flank, Sequencing***
**p7617**	**GTCATTATCTCTACATATACTGAC**	***Sequencing***
**p7618**	**CGTAATTGTGATCCTAATGGTCC**	***Sequencing***
**p7619**	**TCATTGTATCTGATCCAGTCGTC**	***Sequencing***
**p7620**	**ATGTTGTTAGTGGTGTGTATGGTG**	***Sequencing***
**p7621**	**CCAGAACCAAAAGATACAGTACC**	***Sequencing***
**p7622**	**AATCTACCCTTATCAGATGTAGG**	***Sequencing***
**p7623**	**TAAACGGTTGTGTTTGTTTGGTG**	***Sequencing***
**p7624**	**GAGTACCTAATTTCTGTTCTTCTG**	***Sequencing***
**p7870**	**TAGCTTCTGGTAAACCTTCAGC**	***Sequencing***

Table S3: Results of *PfeEF2* sequencing of recrudescent SCID mouse blood samples.

Sample	Cage-Mouse	Treatment	Nucleotide Position	WT Allele	Mutant Allele	Codon Change	Amino Acid Position	WT Amino Acid	Mutant Amino Acid	Note
31	1--1	M5717 12 mg/kg	2258	C	A	ACT → AAT	753	T	N	Mixed
32	1--2	M5717 12 mg/kg	556	T	A	TAT → AAT	186	Y	N	Mutant
33	2--1	M5717 12 mg/kg	556	T	A	TAT → AAT	186	Y	N	Mutant
34	2--2	M5717 12 mg/kg								WT
35	2--3	M5717 12 mg/kg								WT
38	4--1	M5717 12 mg/kg<br>Pyronaridine 12 mg/kg								WT
40	4--3	M5717 12 mg/kg<br>Pyronaridine 12 mg/kg								WT
41	5--1	M5717 12 mg/kg<br>Pyronaridine 6 mg/kg								WT
42	5--2	M5717 12 mg/kg<br>Pyronaridine 6 mg/kg								WT
43	6--1	M5717 12 mg/kg<br>Pyronaridine 6 mg/kg								WT
44	6--2	M5717 12 mg/kg<br>Pyronaridine 6 mg/kg								WT
45	6--3	M5717 12 mg/kg<br>Pyronaridine 6 mg/kg								WT
46	1--1	M5717 12 mg/kg	556	T	A	TAT → AAT	186	Y	N	Mutant
47	1--2	M5717 12 mg/kg	556	T	A	TAT → AAT	186	Y	N	Mutant
48	2--1	M5717 12 mg/kg	556, 2258	T, C	A, A	TAT → AAT,<br>ACT → AAT	186, 753	Y, T	N, N	Mixed
49	2--2	M5717 12 mg/kg								WT
50	2--3	M5717 12 mg/kg	556	T	A	TAT → AAT	186	Y	N	Mutant
56	5--1	M5717 12 mg/kg<br>Pyronaridine 6 mg/kg								WT
59	6--2	M5717 12 mg/kg<br>Pyronaridine 6 mg/kg								WT
RBC Only CTRL	CTRL--3	None								WT

Table S4: Results of eEF2 sequencing of recrudescent samples for the 1x30 mg/kg single oral dose of M5717.

Sample	Nucleotide position	WT allele	Mutant allele	Amino acid position	Codon change	WT amino acid	Mutant amino acid	Parasitemia for the individual mice at blood draw	Recrudescence day post infection
1.1	545	T	C	182	ATT-->ACT	I	T	3.25%	**24**
1.2	413	A	G	138	TAT-->TGT	Y	C	0.50%	**24**

Table S5. Kaplan-Meier curve and log-rank test *p*-value with median estimate for time to recrudescence.

Treatment: M5717 12 mg/kg only
Resistant (n=3) vs no resistant (n=2)
Median time to recrudescence
Resistant: 18 days
Non-resistant: 23.5 days

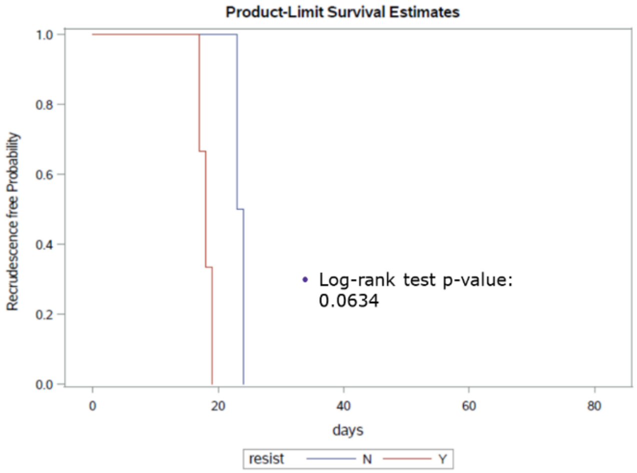

Treatment: M5717 (M) 12 mg/kg only vs M5717 12 mg/kg + Pyronaridine (P) 6 mg/kg
Median time to recrudescence
M5717 only: 19 days
M12+P6: 26 days

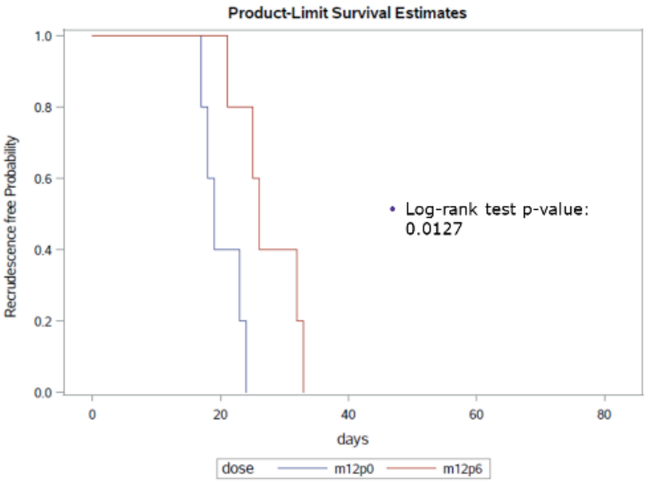

Treatment: M5717 12 mg/kg + Pyronaridine 6 mg/kg vs M5717 (M) 12 mg/kg + Pyronaridine (P) 12 mg/kg
Median time to recrudescence
M12+P6 : 26 days
M12+P12: >52 days

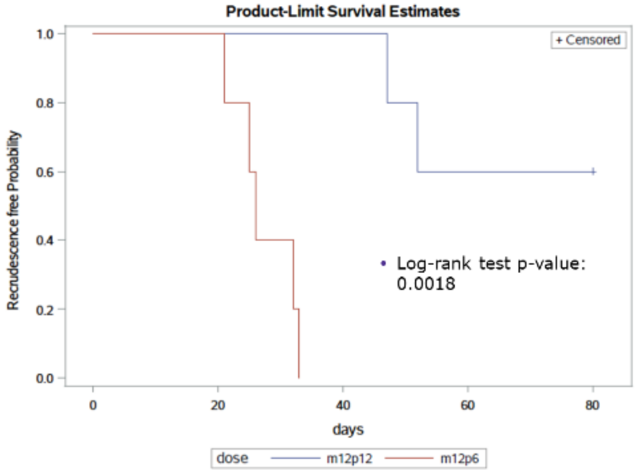

Supplemental References

[i] Desjardins RE, Canfield CJ, Haynes JD, Chulay JD. 1979. Quantitative assessment of antimalarial activity *in vitro* by a semiautomatic microdilution technique. Antimicrob. Agents Chemother.16: 710–718.

[ii] Matile H., Pink JRL. 1990. *Plasmodium falciparum* malaria parasite cultures and their use in immunology. Immunological Methods IV, 221-234 Academia Press.

[iii] Dorn A, Stoffel R, Matile H, Bubendorf A, Ridley RG. Malarial haemozoin/beta-haematin supports haem polymerization in the absence of protein. Nature. 1995, 374: 269-71

[iv] Trager W, Jensen JB. 1976. Human malaria parasites in continuous culture. Science193: 673-5

[v] Huber W, Hurt N, Mshinda H, Jaquet C, Koella JC, Tanner M. 1993. Sensitivity of *Plasmodium falciparum* field-isolates from Tanzania to chloroquine, mefloquine and pyrimethamine during in vitro cultivation. Acta Trop. 52: 313-6.
